# The United Nations sustainable development goals, the WHO general program of work 2019-2023 and international safe communities

**Published:** 2019-08

**Authors:** Dale Hanson

**Affiliations:** ^*a*^Associate Professor, Chair. International Safe Community Certifying Center, Australia.

## Abstract

**Background:**

A number of international safe communities are displaying increasing interest in the United Nations Sustainable Development Goals (UNSDG). The International Safe Communities Certifying Centre is seeking to establish formal relations with the World Health Organization (WHO).

**Purpose:**

A formal review of the UNSDG and WHO General Program of Work 13 – 2019 to 2023 (WHO GPW13) alignment with the international Safe Communities indicators.

**Methods:**

UNSDGs were formally reviewed by 4 senior ISCCC certifiers and ranked in terms alignment with International Safe Communities Indicators.

**Results:**

While injury imposes a considerable cost burden on communities, safety is a critical precondition necessary for the attainment of the productive physical, physical and social environments that communities require to achieve their full potential. Safety provides a dynamic leverage point for communities to achieve their aspiration for sustainable development. There is very strong alignment between the UNSDGs and the International Safe Community Indicators The holistic understanding of injury prevention and safety promotion adopted by the safe communities movement supports the WHO vision to “Promote health, keep the world safe and serve the vulnerable” articulated in its 13th General Program of Work (GPW): 2019 to 2023. Based on the analysis the ISCCC board has identified 7 domains of injury prevention and safety promotion practice that are of high strategic importance8: • Disaster preparedness • Prevention of Road Traffic Injuries • Prevention of Violence against Women • Prevention Violence against Children • Suicide prevention • Falls prevention • Prevention of workplace injury. All new designation applications submitted after the 1st of July 2019 must provide statistical information regarding the incidence of these issues and to describe how they are addressed in their community.

**Conclusions:**

The International Safe Community movement embraces a whole of system (ecological) definition of injury prevention and safety promotion that aligns easily with the United Nations Sustainable Development Goals (UNSDG) and the WHO General Program of Work 13, 2019 to 2023 (WHO GPW13).

**Keywords:**

United Nations SDGs, WHO General Program of Work, ISCCC

